# Supplementary material for: Identification of photoperiod-induced specific miRNAs in the adrenal glands of Sunite sheep (Ovis aries)
Source: Front Vet Sci. 2022 Jul 22;9:888207. doi: 10.3389/fvets.2022.888207 (PMC9354845; doi:10.3389/fvets.2022.888207)
Supplement: Supplementary file 1 [file Data_Sheet_1.ZIP › Supplementary file/miRDeep2 file.zip!/miRDeep2 file/Know_Structure/SPLP42A2/oar-mir-299.pdf]

miRBase precursor : oar-mir-299
Total read count : 258
oar-miR-299-5p read count : 251
oar-miR-299-3p read count : 6
remaining reads : 1

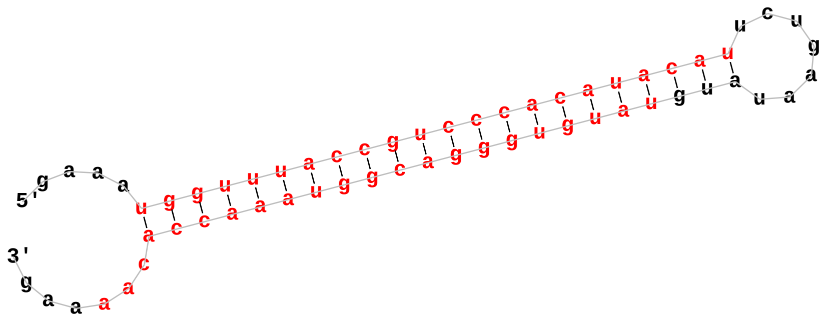

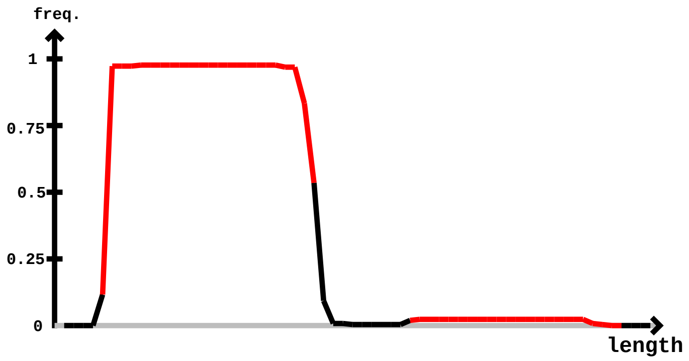

oar-miR-299-5p

oar-miR-299-3p

```
5'- gaaaugguuuaccgucccacauacauucugaauauguaugugggacgguaaaccacaaaag   -3'    exp
    ....((((((((((((((((((((((.......))))))))))))))))))))))......     reads      mm         sample
    ...augguuuaccgucccacauac.....................................     6          0          seq
    ...augguuuaccgucccacauaca....................................     23         0          seq
    ...Gugguuuaccgucccacauaca....................................     1          1          seq
    ....ugguuuaccgucccacau.......................................     2          0          seq
    ....ugguuuaccgucccacauac.....................................     29         0          seq
    ....ugguuuaccgucccacauaca....................................     53         0          seq
    ....ugguuuaccgucccacauacau...................................     114        0          seq
    ....ugguuuaccgucccacauacauu..................................     13         0          seq
    ....ugguuuaccgucccacauacauA..................................     9          1          seq
    ....ugguuuaccgucccacauacauucugaauau..........................     1          0          seq
    .......uuuaccgucccacauacauucu................................     1          0          seq
    ...................................guaugugggacgguaaacc.......     3          0          seq
    ...................................guaugugggacgguaaacca......     1          0          seq
    ...................................guaugugggacgguaaaccaU.....     1          1          seq
    ....................................uaugugggacgguaaacc.......     1          0          seq
```
